# Hearing in a world of light: why, where, and how visual and auditory information are connected by the brain 

**DOI:** 10.16910/jemr.12.7.3

**Published:** 2019-11-25

**Authors:** Jennifer M Groh

**Affiliations:** Center for Cognitive Neuroscience, Duke University, Durham, NC, USA

**Keywords:** Hearing, embodied cognition, interaction between vision and hearing, EMREO, multiplexing, coordinate transformation, reference frame, saccade

## Abstract

Keynote by Jenny Groh (Duke University) at the 20th European Conference on Eye Movement Research (ECEM) in Alicante, 19.8.2019

Information about eye movements with respect to the head is required for reconciling visual and auditory space. This keynote presentation describes recent findings concerning how eye movements affect early auditory processing via motor processes in the ear (eye movement-related eardrum oscillations, or EMREOs). Computational efforts to understand how eye movements are factored in to auditory processing to produce a reference frame aligned with visual space uncovered a second critical issue: sound location is not mapped but is instead rate (meter) coded in the primate brain, unlike visual space. Meter coding would appear to limit the representation of multiple simultaneous sounds. The second part of this presentation concerns how such a meter code could use fluctuating activity patterns to circumvent this limitation

**Video stream** https://vimeo.com/356576513

